# Clinical Outcomes and Patient-Reported Satisfaction with Functional Orthodontic Treatment of Mandibular Condylar Process Fractures

**DOI:** 10.3390/jcm15218401

**Published:** 2026-10-28

**Authors:** Ann Nitzsche, Franziska Krause, Marco Dederichs, Konrad Tolksdorf, Martin Fischer, Stefan Schultze-Mosgau, Collin Jacobs, Christoph-Ludwig Hennig

**Affiliations:** 1 Department of Orthodontics, Center of Dental Medicine, University Hospital Jena, An der alten Post 4, 07743 Jena, Germany; 2 Department of Prosthodontics, Center of Dental Medicine, University Hospital Jena, An der alten Post 4, 07743 Jena, Germany; 3 Department of Oral and Maxillofacial Surgery/Plastic Surgery, University Hospital Jena, Am Klinikum 1, 07747 Jena, Germany; 4 Department of Orthodontics, University Hospital Duesseldorf, Moorenstraße 5, 40225 Duesseldorf, Germany

**Keywords:** mandibular condylar process fractures, collum fracture, functional orthodontic appliances, interdisciplinary patient care team, activator appliances, quality of life

## Abstract

**Objectives:** Mandibular condylar process fracture is the most common fracture of the mandible, with great medical relevance. **Materials and Methods:** In this retrospective exploratory observational study, we describe outcomes in 22 patients after unilateral or bilateral mandibular condylar process fracture. Owing to heterogeneous treatment allocation and the absence of a comparable control group, the findings are descriptive and hypothesis-generating rather than causal. In addition to collecting descriptive data from digital patient records, a survey was conducted using standardized questionnaires on the underrepresented construct of oral health-related quality of life. **Results:** The patient sample comprised 22 individuals between the ages of 8 and 72 years after functional orthodontic or combined surgical–functional orthodontic treatment 2.5 years after sustaining a unilateral or bilateral mandibular condylar process fracture. In this cohort, children showed more favorable observed functional results than adults. Unilateral fractures were associated with better functional outcomes than bilateral fractures. Furthermore, midline shift was more common in unilateral fractures and was associated with functional limitations such as occlusal disorders. **Conclusions:** Both treatment approaches were associated with favorable functional and radiographic outcomes, particularly in children and after unilateral fractures, although these findings should be interpreted as descriptive and hypothesis-generating in light of the limited sample size.

## 1. Introduction

As the most fragile region of the mandible, the mandibular collum is a predilected location for fractures [1,2,3]. The prevalence of mandibular condylar process fracture in adults varies between 17.5% and 50% [4]. Other authors cite the following prevalences: 33.8% [5], 25–30% [6], 30% [7], 52.4% [8], 49% [9], and 41% [10]. Men are more frequently affected than women (75% vs. 25% [8]; 66% vs. 34% [10]). An accumulation is found in the third decade of life in men and in the fourth decade of life in women [4,8,10,11]. The proportion of bilateral fractures is 20% to 33% [4,10]. The causes of fractures are cited as traumas associated with physical altercations, falls, sports injuries, and traffic accidents [8,10,12,13]. In both sexes, occupational accidents are often the cause [4]. In childhood, bicycle accidents (6–12 years of age), followed by traffic accidents (6–18 years of age), and sports accidents (12–18 years of age), as well as falls (1–12 years of age) and maltreatment (1–5 years of age), are cited as causes [14,15]. The change in recreational behavior toward vehicle sports such as mountain biking and skiing as well as increased traffic density have increased the frequency of mandibular condylar process fractures [16].

A mandibular condylar process fracture is defined as a disruption of bony continuity extending from the incisura seminularis mandibulae to, and including, the condylar head [17]. Mandibular condylar process fractures can be classified as high, mid, or low fractures [17]. The force leading to the fracture is predominantly not provoked directly but is trans-mitted [4]. Forces impinging centrally on the chin are transported dorsally via the vertical mandibular branch to the delicate joint parts. If the caput mandibulae strikes against its dorsal boundary, a flexion fracture of the collum mandibulae results if sufficient force is applied [18]. The corresponding distribution of force on both articular processes causes a bilateral fracture [19]. An eccentric, paramedian force more frequently leads to a fracture of the ipsilateral corpus mandibulae and a contralateral unilateral mandibular process fracture [19]. In luxation and dislocation fractures of the mandibular condylar process joint, the physiological traction of the adductors and the simultaneous lack of articular support leads to a craniodorsal displacement of the distal fragment. Simultaneously, the proximal fragment may be displaced anteriorly, medially, and cranially due to traction of the abductors, especially the lateral pterygoid muscle. In contrast to a fracture with dislocation, in a luxated fracture, the condylus remains in the temporomandibular joint socket. The fracture height, degree of dislocation, and course of the fracture line are decisively influenced by localization, direction, the extent of the force applied, and the degree of mouth opening at the time of the trauma [20].

**Figure 1:**
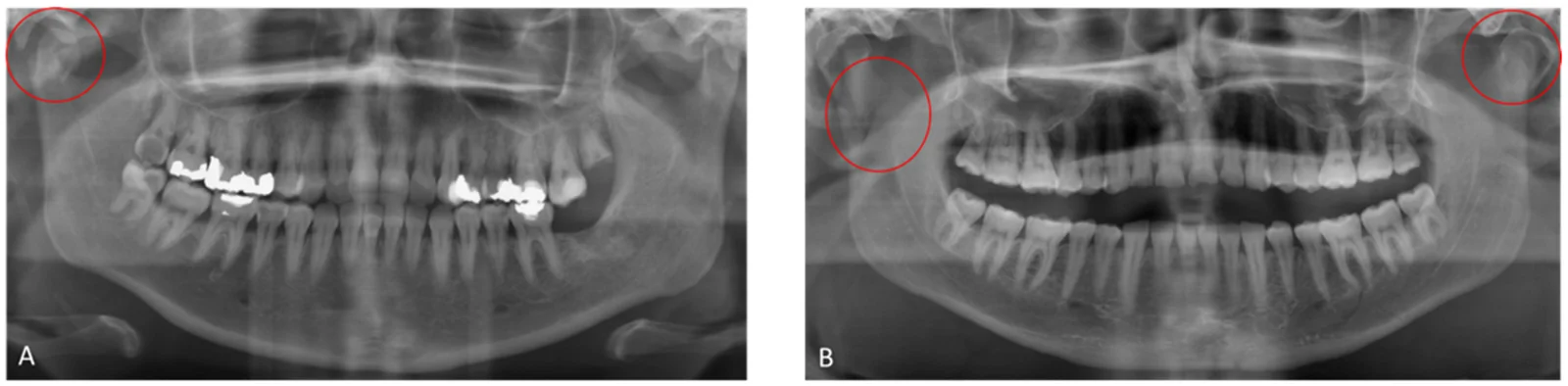
Different types of mandibular condylar process fractures on an orthopantomogram. (**A**) Unilateral high fracture on the right collum and (**B**) bilateral mandibular condylar process fractures, with a low fracture of the right collum and a fracture of the left mandibular condyle head.

Figure 1 shows two different types of mandibular condylar process fractures in an orthopantomogram.

The treatment options for mandibular condylar process fractures are based on four approaches:

1. **Conservative approach;**
2. **Functional orthodontic procedure;**
3. **Surgical procedure;**
4. **Combined interdisciplinary treatment.**

The choice of treatment method is controversially discussed in the literature [21,22,23]. The type of fracture, fracture height and localization, the injury to the joint capsule, the degree of dislocation or luxation, the presence of other mandibular or maxillary fractures, and, most importantly, the patient’s age all influence the therapeutic decision-making process [1,23,24,25,26,27].

In functional orthodontics, the forces of the activated chewing, tongue, cheek, and lip muscles are transferred to the bone, periodontium, and temporomandibular joint. The activator is a removable functional orthodontic treatment appliance. The construction bite relocates the lower jaw into an anterior position. Distal bites support the distraction of the fracture ends. The change in the vertical and sagittal position of the mandible results in altered muscle tone and an altered functional pattern, which initiates ligamentous and osseous remodeling processes [23,24,25,26,27,28]. Restoration of the joint-forming structures is achieved by functional condylar modulation based on dorsocranial growth of the fracture fragments [26]. Functional orthodontic treatment of mandibular condylar process fractures in children has already been the subject of numerous studies [23,26,28,29,30,31,32,33,34,35]. There are only a few studies on mandibular condylar process fractures in adults. A study by Wichelhaus et al., who examined adults with dislocated mandibular condylar process fractures after functional orthodontic treatment using an activator, should be emphasized. Good to very good functional results were achieved in all 14 patient cases. Wichelhaus et al. declared the activator to be a viable treatment option after mandibular condylar process fractures due to its neuromuscular influence potential [36]. Further studies support the therapeutic potential of the functional orthodontic appliance after mandibular condylar process fractures [14,35,36,37].

The aim of the present retrospective study was to determine the influence of age (children vs. adults), fracture type (unilateral vs. bilateral), and midline shift on the functional and morphological treatment outcome, treatment satisfaction, and oral health-related quality of life of patients after mandibular condylar process fractures.

## 2. Materials and Methods

The study included retrospective data from patients who underwent interdisciplinary orthodontic treatment and maxillofacial surgery after a mandibular condylar process fracture between 2015 and 2021. In its notice and statement, the Ethics Committee (file number 2026-4430-Bef) raised no objections to the conduct of the study or the publication of the results. For the retrospective cohort, all patients treated at the University Hospital Jena for a high, medium, or low mandibular condylar process fracture [38] were screened for eligibility and included if they met these criteria. Patients with a complete ramus fracture or a comminuted fracture were excluded because these injuries were managed exclusively by surgical plate osteosynthesis and therefore did not fall within the treatment spectrum investigated in this study. All eligible cases identified during the study period were included in the analysis, resulting in a final sample of 22 patients.

These 22 patients were identified as having undergone surgical, functional orthodontic, or interdisciplinary surgical–functional treatment for a unilateral or bilateral mandibular condylar process fracture.

Indication for activator therapy was considered in cases of non-operable fractures. Combined activator therapy and surgery were used in patients who, in addition to a mandibular condylar process fracture, presented with a fracture of the mandibular corpus that was surgically treated by plate osteosynthesis. Maxillomandibular fixation was applied at the discretion of the responsible oral and maxillofacial surgeon and was used primarily in cases of low and bilateral mandibular condylar process fractures, with or without associated fractures.

Specifically, activator therapy was used in 16 out of 22 patients (72.7%). Combined surgical treatment and activator therapy was performed in 6 patients (27.3%), and maxillomandibular fixation was applied in 8 patients (36.4%). Unilateral fractures were observed in 16 patients (72.7%), while bilateral fractures were present in 6 patients (27.3%).

If a fracture of the mandibular corpus was present, it was treated surgically, and at the discretion of the attending oral and maxillofacial surgeon, subsequently immobilized by maxillomandibular fixation for two weeks. In six patients, maxillomandibular fixation alone was performed at the discretion of the attending surgeon. Once sufficient mouth opening had been achieved, an activator could be inserted approximately one week later. The activator was prescribed for 22 h per day for six months, followed by night-time wear only, for an additional six months. Removal of the osteosynthesis plates was performed six months after surgery. Follow-up examinations were conducted every eight weeks for all treatment modalities. The final examination for all treatment modalities was conducted one year after the fracture occurred.

All patients completed the treatment through the follow-up examination. Compliance can therefore be rated as very high.

### 2.1. Questionnaire on Oral Health and Quality of Life

The patients were subsequently asked to complete a voluntary anonymous questionnaire on oral health and quality of life, treatment satisfaction, and post-interventional symptoms. The survey was conducted once as a cross-sectional study. If participants were under eighteen years of age, the questionnaire was sent to their legal guardians and they filled it in together. By returning the questionnaires anonymously, the patients or their legal guardians consented to participating in the study.

Patients who were fourteen years of age or younger (*n* = 7) at the time of the survey were sent the Child Perceptions Questionnaire (CPQ). According to Bekes [39], the assessment of oral health and quality of life in children, compared to adults, is “a long-neglected topic”. The CPQ has proven to be a suitable and reliable instrument that validly measures the oral health and quality of life in children aged 11 to 14 [40].

It comprises 35 items covering four domains: oral symptoms, functional limitations, emotional well-being, and social well-being. Items are rated on a 5-point Likert scale from 0 (“never”) to 4 (“very often”) and assess how frequently specific events occurred during the preceding three months. The questionnaire also includes two global items on general health and oral health, as well as a question on fixed or removable orthodontic appliances. Higher CPQ scores indicate poorer oral health-related quality of life, whereas lower scores indicate better oral health-related quality of life.

Patients who had turned eighteen received the Oral Health Impact Profile (OHIP) as the oral health and quality of life questionnaire (*n* = 15) [41]. OHIP is an internationally used and validated questionnaire for assessing oral health-related quality of life in adults. It was originally developed by Slade and Spencer in Australia. The abbreviated OHIP-G-14 captures seven domains: functional limitation, physical pain, psychological discomfort, physical disability, psychological disability, social disability, and handicap. Responses are recorded on a 5-point Likert scale ranging from 0 (“never”) to 4 (“very often”). Item scores are summed to yield a total score from 0 to 56, with higher scores indicating greater impairment in oral health-related quality of life.

All instructions were printed on the questionnaire for independent completion. A total of seventeen completed questionnaires were returned, corresponding to a response rate of 77%. Of the 15 adults, 10 (66.7%) responded to the questionnaire. The oral health-related quality of life was separately analyzed in children and adults and evaluated only where questionnaire data were available.

For patient satisfaction, patients were asked about subjectively perceived complaints using a self-assessment questionnaire. The questionnaire recorded the symptoms before and after treatment, as well as the symptoms that were perceived as the most unpleasant. Patients were able to choose between the following: limited mouth function, pain, joint cracking when opening or closing the mouth, altered bite/restriction when chewing, and asymmetries in the face/esthetics. Further complaints could be added under “Other”. Multiple answers were possible. Treatment satisfaction was recorded on a Likert-like scale from 0 (“completely dissatisfied”) to 10 (“completely satisfied”). Patients were asked to rate whether the orthodontic treatment had helped them get better. They were also given the opportunity to assess what would have helped to positively influence the course of the disease. Additionally, they were asked how they felt about wearing the activator.

### 2.2. Measurement of X-Ray Imaging

The medical parameters were extracted from digital patient files with the following data: date of birth, sex, age at event, diagnosis, concomitant injuries, general diseases, cause of fracture, radiographic diagnosis, clinical parameters (midline shift, mouth opening, occlusal disorders, pre- and post-interventional complaints), and type of therapy. Mouth opening was assessed as good at values between 30 and 40 mm and as *restitutio ad integrum* above 40 mm. Lower results were assessed as sufficient functional findings [42]. Collum length was measured retrospectively on digital panoramic radiographs. For this purpose, digital orthopantomograms from 22 patients were retrieved from the PACS system, with one pre-treatment and one post-treatment radiograph per patient. Depending on the side of the mandibular condylar process fracture, measurements were taken 31 times on the right side (*n* = 17 pre-treatment; *n* = 14 post-treatment) and 20 times on the left side (*n* = 10 pre-treatment; *n* = 10 post-treatment).

**Figure 2:**
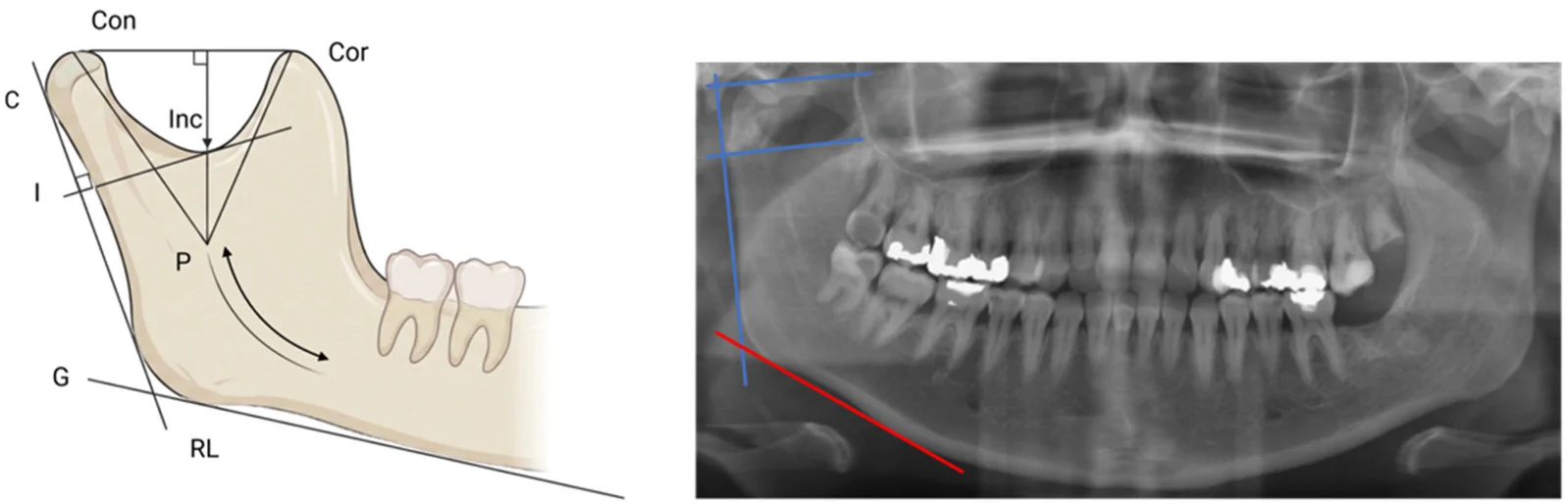
Measurement of ramus height. Con: the most superior point of the condyle; Cor: the most superior point of the coronoid process; Inc: the most inferior point of the mandibular notch; I: the intersection of the RL and a perpendicular line from Inc; C: the intersection of the RL and a perpendicular line from Con; G: the intersection of the RL and ML; RL: a tangent line of the mandibular ramus; and ML: a tangent line of the mandibular body. Modified from Choi et al. 2005 [43].

Two examiners independently assessed the same radiographs in a blinded manner using the predefined landmarks and measurement protocol and measured neck height, ramus height, and total length before and after treatment. Vertical lines were drawn from the highest point of the temporomandibular joint head and the mandibular incisura to the ramus line (Figure 2), and collum height was calculated as the distance between the two interfaces according to the method of Choi et al. [43]. For all analyses, the mean of the two examiner measurements was used. Inter-examiner agreement was evaluated using an absolute-agreement intraclass correlation coefficient approach, with reliability considered for both single measurements and the mean of the two examiners; corresponding 95% confidence intervals were calculated. An increase or no change in overall length was classified as a positive length outcome.

### 2.3. Statistical Analysis

Statistical analyses were performed using SPSS Statistics 27.0 (IBM, Armonk, NY, USA). Descriptive statistics were calculated for all variables. Continuous variables are reported as mean, standard deviation, and range, whereas categorical variables are reported as absolute and relative frequencies. Pearson’s correlation coefficient was used for interval-scaled variables with approximately normal distribution, and Spearman’s rank correlation coefficient was used for ordinal-scaled or non-normally distributed variables. Normality of continuous variables within the compared subgroups was assessed using the Shapiro–Wilk test and graphical inspection of Q–Q plots and histograms. Homogeneity of variances was evaluated using Levene’s test. For two-group comparisons, independent-samples *t*-tests were used when the distributional assumptions were considered sufficiently supported; when equality of variances was not assumed, the unequal-variance form of the *t*-test was applied. Given the small sample size and unequal subgroup distribution, all inferential analyses were regarded as exploratory. All tests were two-sided, with a significance level of *p* < 0.05, and results are reported with exact *p*-values and 95% confidence intervals where applicable.

## 3. Results

### 3.1. Study Population

The patients examined were on average 29 years old (M = 29.1; SD = 18.67; range 8–72).

### 3.2. Medical Parameters

Regardless of the type of therapy, the mouth opening of the total sample increased from an average of 24.2 mm to 42.2 mm by 19.7 mm within the physiological ranges. The increase of 19.7 mm is the result of calculating the mean values for the subgroups (children, adults, unilateral mandibular condylar process fractures, bilateral mandibular condylar process fractures, with maxillomandibular fixation, and without maxillomandibular fixation). Half (*n* = 11, 50%) of the patients had midline shift pre-interventionally.

After treatment (all types of therapy combined), four patients in the total sample continued to have occlusal disorder (*n* = 4; 18.2%). At follow-up, occlusal disorder was no longer documented in 14 patients (63.6%), whereas 4 patients (18.2%) continued to have an occlusal disorder. Only six patients (*n* = 6; 27.3%) were completely symptom-free after treatment. While pre-intervention, reduced mouth opening in combination with pain and an altered bite were perceived as restrictive (*n* = 8; 36.4%), post-intervention, joint cracking and persistent pain were perceived as restrictive. Altered bite and restriction in chewing were perceived as the most unpleasant (*n* = 5; 22.7%). Mandibular condylar process fractures were associated with concomitant fractures in 45.5% of the sample. On average, a high level of treatment satisfaction was reported. Table 1 shows the distribution of the medical parameters of the overall sample.

In addition, there were changes in the length of the collum before and after the intervention (Table 2). Inter-examiner agreement was assessed using a two-way random-effects, absolute-agreement intraclass correlation coefficient; the mean of the two examiners was used for the analyses.

### 3.3. Group Comparisons: Children vs. Adults and Unilateral vs. Bilateral Fractures

The children were on average 12 years old (M = 11.6; SD = 1.9; range 8–14). The fracture occurred 2.5 years prior to the time of the survey (age at time of event: M = 9.1 years; SD = 2.1; range 9–12). Six children suffered a unilateral fracture, and one suffered a bilateral fracture. Five children (*n* = 5; 71.4%) were treated with an activator. Two (*n* = 2; 28.6%) received combined surgical and functional orthodontic treatment. Only two children received maxillomandibular fixation (*n* = 2; 28.6%). According to the questionnaire, all children felt that the activator therapy was helpful.

The adults were on average 37 years old (M = 37.3; SD = 17.2; range 18–72). At the time of the fracture event, the adults were on average 35 years old (M = 34.9; SD = 17.2; range 14–70). Ten adults suffered from a unilateral fracture and five from a bilateral fracture. Eleven adults (*n* = 11; 73.3%) were treated with an activator. Four adults (*n* = 4; 26.7%) received combined surgical and functional orthodontic treatment. Six (*n* = 6; 40%) received maxillomandibular fixation. Of the 15 adults, 10 (66.7%) responded to the questionnaire. Of these 10 respondents, eight (80%) thought that activator therapy was helpful, one (10%) rated functional orthodontic therapy as not helpful, and one (10%) was undecided.

**Figure 3:**
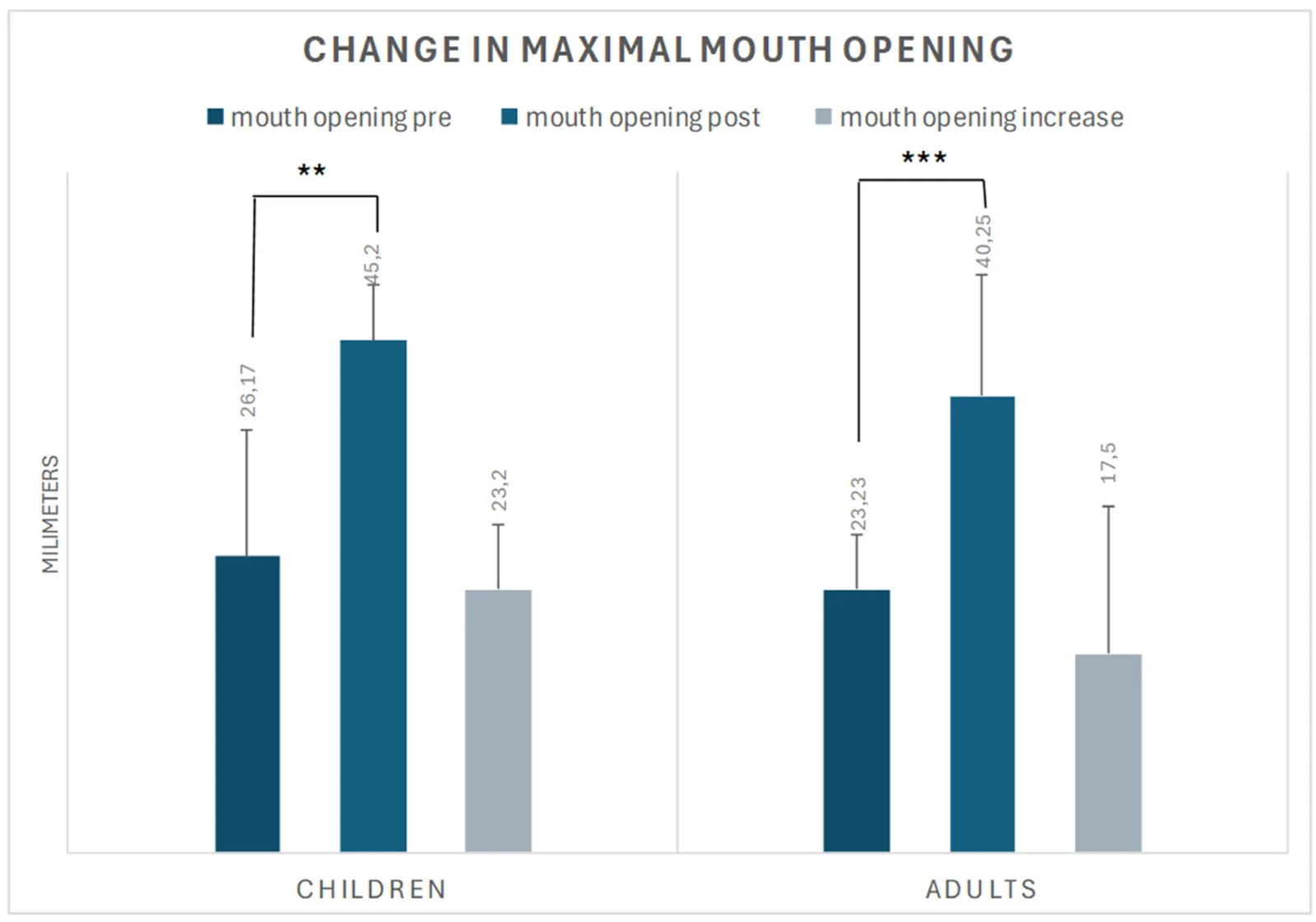
Mean value and standard deviation of mouth opening before (pre) and after (post) therapy as well as the increase in mouth opening in the group comparison: children vs. adults (** = *p* < 0.01; *** = *p* < 0.001).

The mean pre-interventional mouth opening was 26.17 mm in children (SD = 11.13). After therapy, mouth opening was within the normal range in all children (M = 45.2; SD = 4.87). Mouth opening increased by an average of 23.2 mm (SD = 5.76). In adults, mean pre-interventional mouth opening was 23.23 mm (SD = 9.29). After therapy, mouth opening was within the normal range in seven adults (M = 40.25; SD = 10.78). One patient showed a 17 mm increase in mouth opening but did not reach the physiological range even after therapy. Overall, mouth opening increased by an average of 17.5 mm (SD = 13.1) (Figure 3).

**Figure 4:**
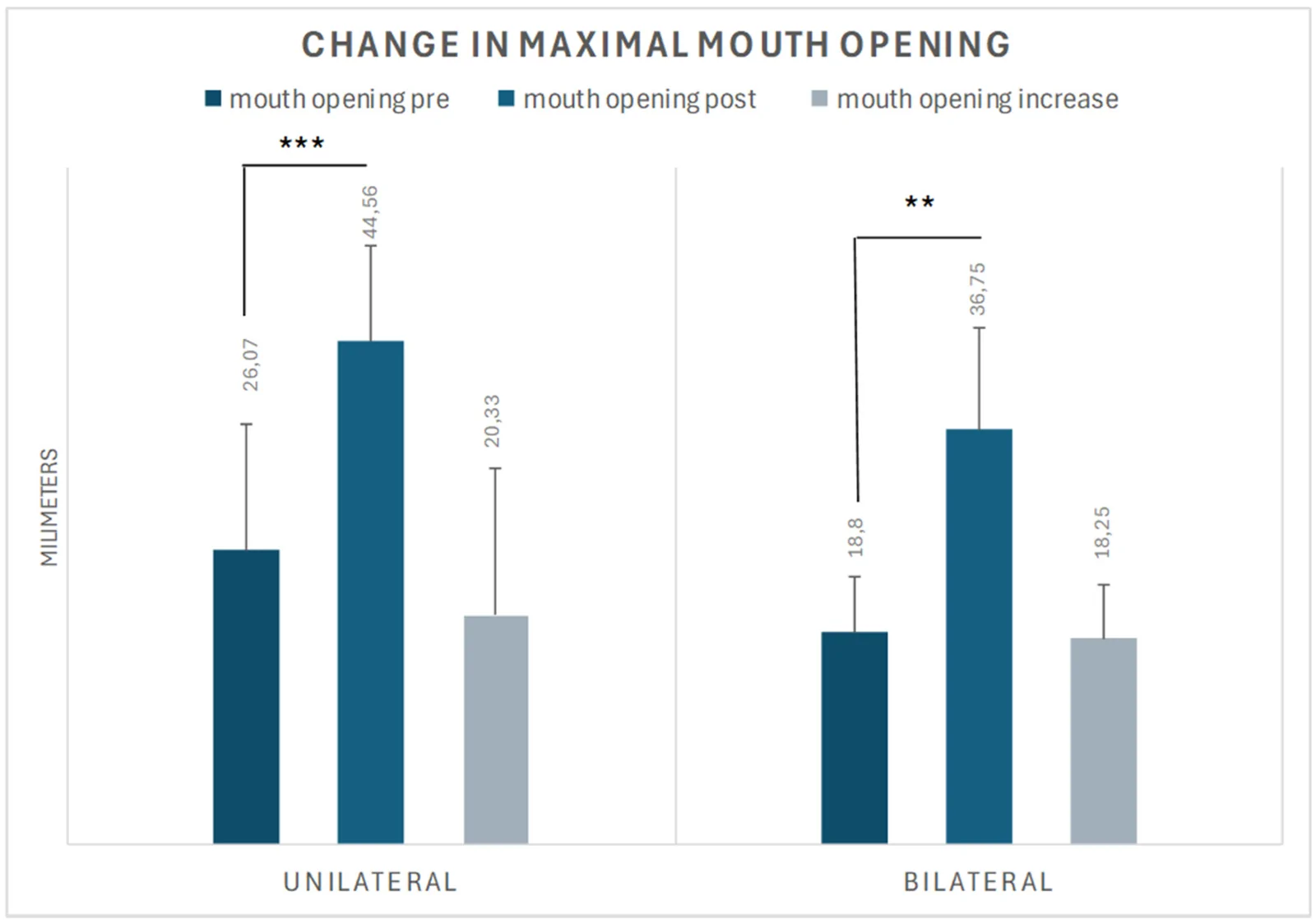
Mean value and standard deviation. Mouth opening before (pre) and after (post) therapy as well as the increase in mouth opening in the group comparison: unilateral vs. bilateral fractures (** = *p* < 0.01; *** = *p* < 0.001).

Mean pre-interventional mouth opening was 26.07 mm in patients with unilateral fractures (SD = 10.02). After treatment, mouth opening was within the normal range in all individuals with unilateral fractures (M = 44.56; SD = 8.46). Mouth opening increased on average by 20 mm (M = 20.33; SD = 12.98). Mean pre-interventional mouth opening was 18.8 mm in patients with bilateral fractures (SD = 6.91). After treatment, mouth opening was in the physiological range at 37 mm (M = 36.75; SD = 8.99). Mouth opening increased on average by 18 mm (M = 18.25; SD = 4.79). Patients with a bilateral fracture showed a more restricted pre-interventional and a lower post-interventional mouth opening as well as a smaller increase in mouth opening compared to patients with a unilateral fracture. Levene’s test did not indicate evidence of unequal variances (*p* = 0.13). Therefore, the equal-variance form of the independent-samples *t*-test was used. The difference in mouth opening increase between patients with unilateral and bilateral fractures was not statistically significant (*t*(11) = 0.31; *p* = 0.77; 95% CI [−12.93 to 17.09]) (Figure 4).

At follow-up, five children (71%) no longer had a documented occlusal disorder. In adults, nine patients (*n* = 9; 60%) were free from occlusal disorders. Post-intervention, no children, but four adults (*n* = 4; 27%) continued to have occlusal disorders. At follow-up, ten patients with unilateral fractures had no documented occlusal disorder (*n* = 10; 62.5%). Of the clientele with bilateral fractures, 67% (*n* = 4) were free of occlusal disorders. Post-intervention, 13% of patients with unilateral fractures and 33% with bilateral fractures continued to suffer from an occlusal disorder. Patients with bilateral fractures were more likely to have concomitant injuries such as lacerations and concomitant fractures than those with unilateral fractures.

There was no significant difference between children and adults in the increase in collum length. The patients with unilateral fractures had higher values for the increase in left collum length (M = 2.8; SD = 2.17) than those with bilateral fractures (M = 1.2; SD = 2.95). A *t*-test for independent samples showed that this difference was not significant (*t*(8.00) = 2.29; *p* = 0.051; 95% CI [(−0.12 to 4.41)]).

### 3.4. Group Comparison for Therapy Satisfaction and Oral Health-Related Quality of Life: Children vs. Adults

On a Likert-like scale of 0 to 10 points, the children selected higher values (M = 9.29; SD = 0.95; range 8–10) compared with the adults (M = 7.1; SD = 3.32; range 1–10). A *t*-test for independent samples showed that this difference was not significant (*t*(15) = 1.97; *p* = 0.068; 95% CI [(−0.58 to 4.96)]). All seven questionnaires of children could be analyzed.

When looking at oral health-related quality of life, the children and adults had to be tested separately. The children surveyed rated their state of health with an average of 2 points (M = 2; SD = 0.82; range 1–3). They rated their oral health slightly lower (M = 2.29; SD = 0.49; range 2–3). In the pediatric subgroup, exploratory Spearman analyses suggested a significant association between perceived oral health status and treatment satisfaction (r = 0.88; *p* = 0.01). A similar association was observed between perceived oral health status and oral health-related quality of life (r = 0.80; *p* = 0.02). Given the very small subgroup size, these findings should be interpreted with caution (Table 3 and Table 4).

The total OHIP score in the sample was 12.8 points (M = 12.8; SD = 14.37). According to Spearman’s correlation, therapy satisfaction and the MLQ (OHIP) were significantly correlated (*p* = 0.01; r = −0.85). The higher the perceived MLQ score on the OHIP (low scores), the higher the therapy satisfaction. Table 5 lists the distribution of the dimensions of the OHIP questionnaire.

## 4. Discussion

Twenty-two patients were included in the study. Adults and children who had mandibular condylar process fractures and were treated with functional orthodontics were examined. Treatment pathways were heterogeneous, including functional orthodontic treatment, surgery, combined surgical–functional management, and maxillomandibular fixation. Because treatment choice was likely influenced by fracture type, localization, degree of dislocation or luxation, associated injuries, and patient age, confounding by indication cannot be excluded. In previous studies, researchers describe that mainly children were treated with functional orthodontics mandibular condylar process fractures. In contrast, the present study also focused on the results in adults. Another difference is the distribution of sexes: the patients described in the literature were predominantly male. Many of the review studies did not provide any information on this [1,25,28,33,36,44,45]. Marker et al. reported a male predominance among patients with mandibular condylar process fractures, with men accounting for 66% of cases [10]. Güven and Keskin also reported a male preponderance in the sex distribution of mandibular condylar process fractures and attributed this to the more impetuous nature of boys [46]. In the present study, the sex-specific distribution was balanced. This may be explained by the higher average age of the sample. Looking at the children separately from the adults, five boys (71%) and two girls (29%) were affected. They had an average age of 9 years at the time of the event (M = 9.1; range 9–12). This average age corresponds to the average age of the patients in our review and is comparable to the sex distributions in studies by Silvennoinen et al. (men 75%, women 25%), Güven and Keskin (boys 61%, girls 39%), and Rampaso et al. (2012) (men 72%, women 28%) [8,45,46].

Statistical analysis of the sample showed that female patients were significantly older than men at the time of fracture. This result is consistent with the literature, which indicates a peak incidence of mandibular condylar process fractures in women between the ages of 30 and 40 years and in men between the ages of 20 and 30 years [4,10,30,47].

The cause of fracture in the sample was predominantly consistent: falls due to syncope, epileptic seizures, or tripping, as well as falls from bicycles, windows, and stairs, were the most common causes of mandibular condylar process fractures. The second and third most common causes, in equal proportions, were car accidents, skiing accidents, and collisions. The sample was not found to contain fracture causes due to rough handling or accidents at work. This result is consistent with the relevant literature, which cites trauma and falls as the main cause of mandibular condylar process fractures [30,37,46,48,49,50,51,52,53]. Only two German studies in our review provided information on the cause of fracture: Wichelhaus et al. also cited falls (bicycle, stairs, and ice) as the most common cause at 42.8% (*n* = 6) [36]. This was followed by road traffic accidents with 35.7% (*n* = 5) and rough play with 14.3% (*n* = 2). Epilepsy was listed separately by Wichelhaus et al. at 7% (*n* = 1) [36].

A study by Pape and Altfeld provided a detailed overview. It cited traffic accidents at 55% (*n* = 78), industrial accidents at 13.5% (*n* = 20), crude offenses at 12.6% (*n*= 18), domestic accidents at 6.7% (*n* = 10), accidents during sports/play at 6.7% (*n* = 10), and epilepsy, collapse, and suicide totaling 4% (*n* = 7) as the main causes of mandibular condylar process fractures [53]. Assessment of the cause of fracture must be evaluated in the context of nationality. While in South Africa, violent crime (86.7%) is the most common cause of temporomandibular joint process fractures, in countries such as Greece, India, China, Italy, and the Netherlands, it is road traffic accidents [12]. For example, a study by Zhou et al. from Wuhan (China) cited car accidents (52%) and motorcycle accidents (16%) as the main causes of mandibular condylar process fractures [13]. Only then do falls follow at 12% [13]. A study by Rampaso et al. in Brazil also cited traffic accidents (44%) followed by falls (39%) and sports accidents and gunshot wounds (5%) as the main causes of mandibular condylar process fractures [46].

In the present study, favorable clinical findings were observed at follow-up. The pre-interventional mouth opening of the sample was significantly reduced. Mouth opening increased following treatment, and post-interventional values were within the physiological range in most patients. Only one patient had reduced mouth opening post-intervention. This was a 50-year-old woman who had suffered a bilateral fracture with concomitant fractures as a result of a fall during a syncope and was treated with a combination of maxillomandibular fixation, surgery, and an activator.

With these results, this study is comparable to a study by Wu et al. [34]. Only in one patient with a bilateral fracture could mouth opening be increased to only 25 mm. Radiographically, severely deformed condyles were observed. In all 13 remaining children, mouth opening changed to a physiological level after maxillomandibular fixation and four weeks of activator therapy [35]. The patient clientele of Wichelhaus et al., at 26.6 years (range 13–38), corresponded most closely to the age spectrum of the present sample. Treatment with maxillomandibular fixation and spring activator significantly increased the mouth opening of 14 patients with a dislocated mandibular condylar process fracture from an average of 25 mm by 15.1 mm to 47.3 mm [36].

Furthermore, in this study, there was no clear pattern in the increase in collum length, and the groups did not differ significantly. In addition, the panoramic imaging is susceptible to magnification, geometric distortion, and patient-positioning variability; consequently, these measurements in a 2D X-ray should be interpreted cautiously. Given the small mean radiographic changes observed, these reliability estimates are relevant for judging whether the measured collum-length differences are likely to exceed measurement variability. The radiographic results should therefore be regarded as exploratory rather than definitive evidence of remodeling.

Studies have repeatedly demonstrated the great morphological remodeling potential of children’s bones compared to those of adolescents and adults [26,29,31,34,35,37,50,52,53,54,55]. A predominantly morphological restoration of structures close to the temporomandibular joint has been described in children. Boffano et al. confirmed the enormous remodeling potential of children’s temporomandibular joint structures up to the age of 13 years [33]. Other authors mention an age of less than 12 years [50] or up to 11 years [29]. According to Kahl-Nieke and Fischbach, over-10-year-olds show a significantly greater dispersion and difference in the mediolateral and anteroposterior condyle diameter, which was interpreted as a lower remodeling potential [27]. Strobl et al. demonstrated complete remodeling in the 2- to 6-year-old age group [37]. The condyles of 7- to 10-year-old children were incompletely restored. The spectrum ranged from minor to hypertrophic condylar deformity and reduced collum neck height [37]. Adolescents appear to have the same capacity for extensive new bone formation, but they lack the capacity for bone resorption, which is no less important for sufficient remodeling [31]. Remodeling in adulthood corresponds to a functional adaptation. It does not result in the formation of a new condyle [46]. Since bone growth is complete, the ability to remodel is severely limited. In order to achieve optimal anatomical alignment, supportive surgical intervention is often necessary in post-pubertal age [50]. The period for remodeling processes can be observed up to 15 years after trauma. The success of radiological therapy then depends on the time of the follow-up examination [31,56,57,58].

Children and adults differ in terms of medical parameters, collum length increase, and treatment satisfaction. Any subgroup differences observed between children and adults or between unilateral and bilateral fractures should thus be interpreted with considerable caution. The overall sample was small (*n* = 22), and the subgroup analyses were based on very small cell sizes, particularly in the pediatric cohort (*n* = 7). In addition, fracture distribution differed substantially by age, as only one of the seven children presented with a bilateral fracture, whereas bilateral fractures were more frequent in adults, creating substantial confounding between age and fracture type. Under these conditions, subgroup comparisons are highly unstable and do not allow firm conclusions about whether observed differences are attributable to age, fracture pattern, or their uneven distribution across treatment groups. The findings are therefore best regarded as descriptive and exploratory, and should be confirmed in larger samples with clinically meaningful stratification or separate analyses for children and adults.

Children in this study showed more favorable observed functional outcomes and higher treatment satisfaction than adults in this cohort, but these subgroup differences cannot be attributed to age alone. The groups differed not only by age but also by fracture distribution and treatment characteristics, and adults more often presented with bilateral fractures, which are associated with less favorable functional findings and more persistent occlusal problems in this sample. Given the small overall sample and the very small pediatric subgroup, these comparisons are best interpreted as descriptive and exploratory, not as evidence of an independent age effect.

Post-intervention, the mouth opening of all children was in the physiological range. A larger increase in mouth opening was observed in children than in adults in this cohort. None of the children showed post-interventional occlusal disorders. At follow-up, occlusal disorders remained documented in about a quarter of adults. When evaluating these results, it must be considered that the adults suffered bilateral fractures more frequently. In the literature, bilateral fractures are associated with an increased risk of malocclusion [34,42,54].

An increased risk of malocclusion is also associated with midline shift. In the present sample, 50% of the patients had midline shift before treatment. In the literature, the prevalence of midline shift after mandibular condylar process fracture is given as 45.9% [53], 50% [28], 42% [26], 57% [49], 21.4% [33], and up to 100% [57].

This finding may be related to the distribution of unilateral and bilateral fractures in the two groups, as the children in the present sample more frequently had unilateral fractures. Studies have shown a correlation between unilateral fractures and the presence of midline shift [34]. The present study is comparable to the one by Holtgrave et al., where mouth opening and occlusion were undisturbed post-interventionally in all 18 children studied. There was no facial asymmetry, pain, or cracking on mouth opening. Half of the children showed a deviation of the mandible to the fractured side when opening the mouth, and the authors verified the shortening of the collum height as the cause [28].

Boffano et al. also demonstrated complete clinical and functional recovery in all children examined [33]. Malocclusion was not observed in any child. The results are supported by Nørholt et al. [55]. They divided 55 patients between the ages of 2 and 20 years after mandibular condylar process fractures into five age groups. They showed that the values of the dysfunction index increased significantly with age. In the younger age groups, there were significantly more patients who showed no clinical signs of dysfunction [55]. Dahlström et al. also found the anatomical and functional restitution of the temporomandibular joint to be lower in adolescents than in children [31]. However, this rarely led to objective symptoms. In the adult group, signs of dysfunction were frequently observed, but these were not rated as severe by the patients. Although fractures were more severe in the youngest age group, dysfunction was almost twice as common in the oldest group [31,56,57]. Altmann and Gundlach demonstrated significantly better clinical outcomes in children under the age of 8 years than in their older counterparts [32]. In addition to age, the type of fracture played a decisive role in the severity of functional disorders. Wichelhaus et al. were able to demonstrate good to very good functional rehabilitation in 14 patients with an average age of 26.6 years [36]. They also rated functional rehabilitation in adults as less favorable. Recent evidence further supports that open rigid internal fixation, when combined with adequate postoperative physiotherapy, can result in substantial recovery of temporomandibular joint function after condylar fractures [59].

In the present study, the patients in the sample rated their satisfaction level with the treatment as high. In this study, questionnaire completion rates should be reported separately for children and adults, and possible non-response bias should be acknowledged, particularly because only 10 out of 15 adults responded. The participants of other studies also reported high levels of treatment satisfaction [26,33,51,53]. Unfortunately, none of those studies revealed how treatment satisfaction was assessed.

Children also reported higher treatment satisfaction than adults, but this finding should likewise be interpreted as descriptive because the groups differed not only by age but also by fracture pattern and treatment characteristics. Treatment satisfaction was significantly related to perceived oral health-related quality of life. Lower perceived oral health was associated with lower treatment satisfaction and lower oral health-related quality of life. The sample was more burdened in terms of oral health-related quality of life than a population-representative sample [39]: 70% of the general population had a higher oral health-related quality of life than the patients studied. The sample was also more burdened than 80% of the general population without orthodontic treatment and more burdened than 50% of the patients in the population-representative sample with orthodontic treatment. Bekes et al. demonstrated a significant influence of orthodontic treatment on the oral health-related quality of life [39]. Children were particularly affected by the functional limitation subscale [58]. Importantly, adults had a higher proportion of bilateral fractures, whereas most children had unilateral fractures, creating substantial confounding between age and fracture type.

A previous study also reported a significant correlation between treatment satisfaction and oral health-related quality of life in adults [41]. Beyond patient-reported outcomes, Su et al. showed that condylar involvement in mandibular fractures was associated with a substantially increased risk of postfracture radiological temporomandibular joint degeneration [60]. Postoperative temporomandibular dysfunction may still occur after mandibular fractures not involving the condyle, although most patients remain symptom-free at six months [61]. Adults after mandibular condylar process fractures reported poorer oral health-related quality of life than 90% of the representative population sample with natural teeth and no removable dentures, 70% of the population sample with natural teeth and removable dentures, and 60% of the edentulous population with complete dentures [41,62].

It was noticeable that children felt particularly impaired in functional aspects such as mouth breathing, slurred speech, and chewing, but also oral symptoms such as bad breath and sores. Adults were predominantly mentally stressed, unable to relax, and felt insecure and embarrassed. Mandibular condylar process fractures affect not only physical, but also psychological, social, and functional areas of life. This is reflected in the greatly reduced oral health-related quality of life of the patients compared to the representative population sample. This should not be equated with treatment satisfaction. While children primarily mentioned functional problems, adults placed psychological impairment in the foreground.

Overall, the interpretation of these results is limited by the retrospective design, the small sample size, the heterogeneity of treatment pathways, and the possibility of selection and indication bias, as treatment allocation was not standardized but likely influenced by fracture characteristics, associated injuries, and patient age.

Further studies, particularly prospective studies involving adults, are desirable to improve our understanding. In the future, children and adults should be strictly separated or analyzed in clinically meaningful age groups.

## 5. Conclusions

In this retrospective cohort, favorable functional, radiographic, and patient-reported outcomes were observed following functional orthodontic treatment alone or combined surgical–functional management. Given the absence of a control group and the non-standardized treatment allocation, these findings are best interpreted as descriptive and hypothesis-generating rather than as evidence of treatment efficacy. Within this exploratory framework, the findings are consistent with the possibility that children, owing to generally greater skeletal remodeling capacity, may have greater potential for functional recovery and morphologic adaptation than adults. However, the observed age- and fracture-related differences should be interpreted with considerable caution, as the small sample size and the unequal distribution of unilateral and bilateral fractures across age groups do not allow for a robust separation of age effects from fracture-type effects. At the same time, the available literature and the present findings suggest that adults may also achieve meaningful functional and structural improvement when functional orthodontic therapy is carefully indicated, although outcomes may be more variable and may be influenced by fracture patterns, baseline severity, and the more limited remodeling capacity in adulthood. Larger, adequately stratified studies are needed to determine whether functional and morphologic outcomes truly differ according to age, fracture type, or both.

## Figures and Tables

**Table 1:** Distribution of the medical parameters of the total sample, mean (M), standard deviation (SD), range, and number (*n*).

Variable	Total
Mouth opening pre-intervention (mm)	M = 24.2; SD = 9.7; range 7–47
Mouth opening post-intervention (mm)	M = 42.2; SD = 9.1; range 24–58
Mouth opening increase (mm)	M = 19.7; SD = 10.9; range 0–37
Midline shift pre-intervention	*n* = 11 (50%)
Associated trauma	Small laceration-squeezing wound *n* = 7 (31.8%)
	Big laceration-squeezing wound + *n* = 12 (54.5%) Polytrauma *n* = 1 (4.5%)
Pre-interventional occlusion disorder	*n* = 18 (81.8%)
Post-interventional occlusion disorder	*n* = 4 (18.2%)
Concomitant fracture	*n* = 10 (45.5%)
Satisfaction	M = 8; SD = 2.78; range 1–10

**Table 2:** Collum lengths on the right and left and their increases in the total sample; mean (M), standard deviation (SD), range, number (*n*), and development of collum lengths.

Variable	Total
Collum length right pre (mm)	M = 23.18; SD = 5.39; range 15–32; *n* = 17
Collum length right post (mm)	M = 23.93; SD = 5.34; range 17–34; *n* = 14
Collum length right increase (mm)	M = 0.86; SD = 2.77; range −5–6; *n* = 14
Collum length right increased	*n* = 8 (57%)
Collum length right taken down	*n* = 4 (29%)
Collum length right equal	*n* = 2 (14%)
Collum length left pre (mm)	M = 18.5; SD = 5.21; range 11–27; *n* = 10
Collum length left post (mm)	M = 20; SD = 5.14; range 10–27; *n* = 10
Collum length left increase (mm)	M = 1.5; SD = 1.84; range −1–4; *n* = 10
Collum length left increased	*n* = 5 (50%)
Collum length left taken down	*n* = 1 (10%)
Collum length left equal	*n* = 4 (40%)

**Table 3:** Distribution of health status and oral health status in children after functional orthodontic treatment of mandibular condylar process fractures.

	State of Health *n* (%)	Oral Health Status *n* (%)
Excellent	2 (28.6%)	0 (0%)
Very good	3 (42.9%)	5 (71.4%)
Good	2 (28.6%)	2 (28.6%)
Mediocre	0 (0.0%)	0 (0%)
Bad	0 (0.0%)	0 (0%)

**Table 4:** Dimensions of the CPQ in children, mean (M), and standard deviation (SD).

CPQ Dimension	Total (*n* = 7)
Oral Symptoms	M = 4.29; SD = 2.29
Functional Limitation	M = 5.14; SD = 4.22
Emotional Well-Being	M = 0.71; SD = 1.49
Social Well-Being	M = 3.00; SD = 3.92

**Table 5:** Dimensions of the OHIP in adults, mean values (M), and standard deviation (SD).

OHIP Dimension	Total (*n* = 10)
Functional Limitation	M = 1.22; SD = 1.99
Physical Pain	M = 2.24; SD = 2.35
Psychological Discomfort	M = 2.23; SD = 2.74
Physical Disability	M = 1.61; SD = 2.22
Psychological Disability	M = 2.74; SD = 2.91
Social Disability	M = 1.81; SD = 2.15
Handicap	M = 1.13; SD = 1.45

## Data Availability

The original contributions presented in this study are included in the article. Further inquiries can be directed to the corresponding author.
